# Introduction to the Special Issue: Social Control, Housing and the Law

**DOI:** 10.1177/09646639251329809

**Published:** 2025-04-15

**Authors:** Lieneke Slingenberg, Michel Vols

**Affiliations:** 1190 Vrije Universiteit Amsterdam, the Netherlands; 3647 University of Groningen, the Netherlands

**Keywords:** social control, housing, homelessness, right to housing, eviction

## Abstract

In this special issue on housing and social control, we explore the relationship between these two concepts through a socio-legal perspective. We investigate how law plays a role in shaping and assessing the connection between housing and social control, by, on the one hand, serving as an instrument of social control, and, on the other hand, acting as a limitation on the ability to impose social control. In addition to this dual function of the law, we identify four scenarios where social control and housing intersect, ranging from the complete absence of housing, to access to, occupation of and eviction from housing. The contributions to the special issue have a wide geographical scope (including case studies from Europe, America, Africa and Australia); each deals with one or more of these four scenarios, and they each underline one or both of the two functions of law as regards social control.

## Introduction

This special issue delves into the analysis of housing and social control through a socio-legal lens. Before we embark on exploring the relationship between these two concepts, it is crucial to understand their meanings. Housing, in its essence, refers to the place where people live, reside, and dwell. The concepts of housing, house, and home have been extensively discussed in various disciplines (Clapham, 2005; Després, 1991; Sennet, 2018). Fox O'Mahony (2007) has delved into the meaning of house and home in sociolegal scholarship, describing the home as a house plus an x-factor. The house represents the physical structure, while the x-factor embodies the social, psychological, emotional, and cultural significance of this structure for its occupiers. This links to the legal interpretation of the concept of ‘home’ by, for example, the European Court of Human Rights. In its standard phrase the Court stresses that ‘[w]hether or not a particular premises constitutes a “home” (…) will depend on the factual circumstances, namely, the existence of sufficient and continuous links with a specific place’ (*Winterstein and others v. France,* para. 141 with further references). Accordingly, the concept of ‘home’ is not limited to the property of which an individual is an owner or official tenant; it refers to the place where somebody factually lives, either lawful or unlawful, and has established certainties. Of course, the question when sufficient ties are established, or when a place has enough significance for its occupiers to call it a home, remains open for contestation. In a recent article, socio-legal scholar Meers (2023) explains why home could even be seen as an essentially contested concept. In line with this, we acknowledge that the concept of ‘home’ is open to many alternative interpretations. The different contributions of this special issue will illustrate this.

The concept of social control has been extensively dissected in the literature as well. Chriss (2022), for instance, sees it as the array of mechanisms, strategies, and institutions that societies employ to regulate individual behaviour and uphold social order. He underscores that these mechanisms can be either formal, such as laws and regulations, or informal, such as norms and customs. Cohen (1985) perceives social control as the practices, institutions, and mechanisms that steer behaviour, deter deviance, and promote adherence to societal norms and values. As Johnsen et al. (2018) argue, the concept of social control is closely related to the concept of power. Power comes in many forms, ranging from force and coercion to influence and bargaining. Johnsen, Fitzpatrick and Watts underline, however, that more ‘softer’ forms of social control are not necessarily less problematic; each mode of social control raises its own moral and practical dilemmas. The contributions in this special issue focus particularly on the legal dilemmas caused by different forms of social control.

## Relationship Between Housing and Social Control

This special issue does not aim to redefine the concepts of social control and housing but rather to dissect the relationship between the two through a socio-legal lens. Previous research has touched upon this relationship (e.g., Johnsen et al., 2018), but this special issue goes a step further, identifying at least four scenarios where social control and housing intersect (cf. Vols, 2024). These scenarios, while not rigidly defined, serve as a framework that better comprehends instances of social control within the housing context.

The first scenario deals with the *absence* of housing, with regulating and controlling people who do not have housing and live on the street (Arundel & Lennarts, 2020). Of course, the stigma and social exclusion of homeless people can be seen as a form of social control itself (Belcher & DeForge, 2012; Kim et al., 2023; Rayburn & Guittar, 2013). Yet, more explicit forms of social control can be characterised as the criminalisation of homelessness. Research worldwide and a report by United Nations Special Rapporteurs have shown that national legislators and local authorities have banned begging or panhandling, sleeping on the street, and loitering (Schipaanboord & Vols, 2024; Special Rapporteur on extreme poverty and human rights & Special Rapporteur on adequate housing, 2024; Walsh et al., 2024).

Besides that, private property owners ban homeless people from properties, including semi-public places such as shopping malls, parks, and streets (Doherty et al., 2002; Mitchell, 2020). Both governments and private property owners have used hostile architecture to prevent homeless persons from sitting, sleeping, and meeting on the streets (Johnsen et al., 2018; Petty, 2016). Informal settlements where homeless people live and sleep are destroyed, moving people experiencing homelessness from one place to another (Blomley et al., 2020; Przybylinski, 2024; Slingenberg & Bonneau, 2017).

The second scenario involves individuals seeking *access* to housing. This group includes people who find themselves completely outside of the housing market and system, such as homeless individuals, persons released from prison and migrants arriving in a new country (Van Tongeren, 2022). Others are already part of the housing market/system as they already have a home. However, they may need to access different housing due to life events, inadequate current housing, others requiring them to vacate their dwelling or a desire to move to a new location (Pastrana & Vols, 2024)

Access to housing is often hindered by barriers that serve to exert social control. Pastrana and Vols (2024) have identified several barriers that can physically prevent individuals from entering or using housing. Such obstacles may stem from how buildings are constructed or the location of housing, leading to inadequate access. These issues could result from oversight by architects or urban planners, but they can also be deliberately implemented as a form of social control. For instance, deliberately confining Roma campsites to polluted areas without access to any resources (Marinaro, 2014).

Individuals may also create barriers to accessing housing, which can be considered legitimate or illegitimate forms of social control. For example, national tenancy laws often give landlords the right to refuse to lease to tenants who have previously failed to pay rent. Additionally, research indicates instances of landlords practising racial or gender discrimination when selecting tenants (Ghekiere et al., 2023; Gusciute et al., 2022).

Barriers to accessing housing are often institutional or systemic. Governmental policies and market outcomes can sometimes favour specific social groups in the context of housing, which can enhance social control. For example, tax benefits may make owning housing more attractive than renting housing (Kholodilin et al., 2023). Governments may intentionally reduce public housing to undermine their political opponents (Chou & Dancygier, 2021; Clegg, 2016). Zoning and planning laws may implicitly exclude certain groups from housing in specific areas (Rothstein, 2017). Banking policies, as seen in the case of redlining, may also prevent people from accessing housing (Swope et al., 2022). Governments may intentionally want to exclude underprivileged people from accessing housing in specific areas due to safety concerns, such as with the Dutch Rotterdam Act (Van Gent et al., 2018; Van Tongeren & Vols, 2017; Vols, 2022). The lack of affordable housing in many countries is another systemic barrier. Government and market failures to address this housing shortage may also be seen as social control since the propertyless and other vulnerable communities could be further marginalised.

In the third scenario, the intersection of housing and social control involves the *occupation* of a home. The architecture of a building itself can reflect social control (Garland et al., 2018). Additionally, enforcing building codes can impose social control on the owners and residents of houses (Bartram, 2022; Vols & Belloir, 2019). Rules imposed by governments, landlords, or condominium associations may restrict individuals’ freedom to do as they please in their homes. For instance, landlords may prohibit tenants from having pets or inviting guests (Boesveldt & Loomans, 2024; Flint & Pawson, 2009). Individuals with mental health issues may have to comply with intrusive requirements in their homes, such as the constant threat of unannounced visits by caregivers (Vols, 2022), (irregular) migrants may have to comply with geographical restrictions, curfews and daily reporting obligations (Slingenberg, 2020, 2021), and homeless individuals may be subjected to curfews in shelters or are required to participate in religious activities (McJunkin, 2023; Tsai et al., 2024; Versteeg et al., 2025). In some countries, public landlords even have a police force that may search residents and their visitors in the corridors of the housing complex (Braga et al., 2024).

The fourth scenario deals with *evictions* from homes. Evictions are a well-known tool for social control (Bruijn & Vols, 2018; Desmond & Valdez, 2012). Landlords use the threat of eviction to make residents comply with their payment and behaviour obligations (Buhler, 2024; Flint & Pawson, 2009; Vols, 2019; Vols, 2023). Governments may use eviction to tackle crime and anti-social behaviour (Bruijn et al., 2018). Eviction is the first tool to tackle squatters and other illegal occupiers (Vasudevan, 2023). There are also examples where evictions were used to enforce discriminatory policies. For example, evictions were used by the South African Apartheid regime to disperse people of colour (Strauss & Liebenberg, 2014; Vols & Fick, 2017). Homeless individuals may be banned from shelters because they violate the rules of the shelter (Kerman et al., 2024). In wars, mass evictions are often used as a weapon causing domicide (Akesson & Basso, 2022). Many evictions are problematic in light of the offered legal protection; some have even called for the abolition of evictions (Bowman, 2024).

The contributions in this special issue each deal with one or more of these four scenarios; ensuring a broad analysis of the relation between housing policies and social control.

## A Legal Analysis

In this special issue, we approach these four scenarios mentioned above from a legal perspective. Instead of taking a formalist approach, we examine the role of law in constructing and assessing the relationship between housing and social control. Law has different relations to the phenomenon of social control. On the one hand, law is an *instrument* of social control. It enables, produces and facilitates different forms of social control. Many agree that social control is a major or even the main function of law, as it functions as an instrument of constraint to keep people from bad behaviour (Fuller, 1975). Law is then often seen as a special form of social control, as an institutional, governmental and/or authoritative form of social control (Griffiths, 2017). This has even been found true for human rights law, which is generally considered to protect individuals against state-sanctioned forms of social control (Johnson and Falcetta, 2021). On the other hand, law can function as a *constraint* on the power of the state to exercise social control and as *protection* against social control exercised by others. This is especially true for law that is developed and located somehow outside of the purview of the government, such as common law, customary law (see also Fuller, 1975), constitutional law or supranational law (Slingenberg, 2020). In this way, law can also limit the use or the effect of the exercise of social control. Both these functions of the law as regards social control will be addressed by the contributions of this special issue.

## Contributions to the Special Issue

Our special issue seeks to deepen our understanding of the relationship between housing and social control through a socio-legal lens. Contributions to the special issue often problematise instances of social control from a critical, legal perspective. The contributions have a diverse geographical scope, including case studies in Canada, Spain, Greece, South Africa and Australia. They each deal with one or more of the four scenarios of housing and social control discussed above, and they each stress or analyse one or both of the two different functions of law as regards social control discussed before.

*Alexandra* *Flynn's* (2025) contribution is about the eviction of encampments in Canadian cities. She explains how the lack of investment in affordable and social housing has resulted in an increase in the number of people experiencing homelessness in Canadian cities. One of the consequences of this increase is the establishment of informal settlements, often in tents and makeshift shelters (‘encampments’) within cities. In her contribution, Flynn analyses recent Canadian case law in relation to the eviction of such encampments. In this case law, courts have set the parameters for the regulation of public space and encampments. Flynn shows how a recent judicial decision challenges the presumption that the availability of any shelter is sufficient to justify encampment evictions. This decision brought attention to the standards within shelters, not simply their blanket availability, and in so doing, acknowledged that as a form of social control, shelters require scrutiny. Even though the case law discussed in this contribution is about evictions, Flynn clearly discusses this within the frame of the first scenario described above, that of the *absence* of housing. For Flynn, homeless means the absence of available, secure or adequate housing, which means that the makeshift encampments do not qualify as homes (see for a different approach Slingenberg and Bonneau, 2017). This contribution clearly shows how the law can *constrain* the use of social control.

Similarly, *Thalia Anthony and Jessie Hohmann* (2025) discuss in their contribution how court rulings can *constrain* the social control exercised by the government in housing policies. Their contribution deals with the third scenario of the intersection of housing and social control, the *occupation* of homes. Their specific case study concerns the dire housing conditions of residents of Ltyentye Apurte, a remote Aboriginal community in Australia. Anthony and Hohmann show how residents of this community had to pursue their rights through legal strategies, and they analyse this through the lens of Aileen Moreton-Robinson's ‘white possessive logics’. In this way, they complement the current literature on housing and social control by illuminating the colonial, racialised and Indigenous context of certain housing policies. Anthony and Hohmann argue that even though adjudication by the courts can provide basic rights to humane housing, courts are largely unable to push back on the ‘white possessive logics’ and often cement and protect these logics through legal narratives.

Other contributions also focus on the shortcomings of the law in effectively *constraining* or countering social control exercised by states in housing policies. *Serde Atalay* (2025) analyses in her contribution the case law of the European Court of Human Rights in cases concerning houses. Through an analysis of the case law on the definitional scope of Article 8 of the European Convention on Human Rights regarding housing and on the application of Article 8 in public eviction cases, she establishes a protection asymmetry. In dealing with both the second (*access to*) and the fourth (*eviction from*) housing scenario, Atelay's analysis shows a protection asymmetry between, first, the possessed and the dispossessed, and, second, between lawful and unlawful occupiers. She concludes that the relation of Article 8 to housing and social control paints a rather bleak picture for those who most need and would most benefit from the protection of Article 8.

Likewise, *Lieneke Slingenberg* underlines in her contribution the limited, or even absence of, protection provided by human rights law as regards social control included in state housing policies. Her case study entails the third housing scenario (*occupation*): the conditions in the new reception and identification centres for asylum seekers on the Greek islands. Since these centres are remotely located, highly surveilled and residents are subjected to a significant curfew measure, Slingenberg shows how the residents generally experience these centres as prisons. These centres would, however, not be qualified as involving a full ‘deprivation of liberty’ under current human rights law. In addition to establishing this limitation in human rights law, she uses Elizabeth Ashford's notion of ‘subsistence exchange contracts’ to argue that the containment measures in the centres could and should be recognised as a deprivation of liberty under human rights law. This would bridge the gap between asylum seekers’ lived experiences and the law and more effectively constrain state power.

Other contributions address how law can be an *instrument* of social control in housing policies. Both Leroy and Ruiz Ramos focus on the social control employed in the reception of asylum seekers in the Spanish enclaves Ceuta and Melilla, but while Leroy addresses the third (*occupation*) and fourth (*eviction*) housing scenario, by focusing on the internal rules for people living in a reception centre, and the social control exercised through the threat of eviction, Ruiz Ramos addresses the first scenario (*absence*) by focusing on the right to choose a place of residence. *Isabella Leroy* (2025) analyses *how* social control is legally exerted in the Centro de Estancia Temporal de Inmigrantes (Temporary Reception Centre for Immigrants, ‘CETI’) of Melilla. Instead of focusing (solely) on the content of the norms that regulate this centre, she focuses on the nature of the norms. Her analysis reveals that the reception centre is primarily governed by a ‘patch-work of rapidly evolving soft law emanating primarily from the executive branch’. In this way, she argues, social control is further facilitated through the nature of the law by granting local authorities a high degree of discretion over the residents of the centre. Accordingly, coercive forms of social control can be made effective not only through the content of the laws regulating accommodation themselves but also in the legal uncertainty created by the nature of those norms, which has material consequences for those living in the CETI.

*Juan Ruiz Ramos* (2025) also analyses how social control is enforced by law. He refers to the concept of legal social control. This refers to the situation where social control is enforced by legal institutions through norms established in law. While this form of social control is often positively evaluated, his contribution shows the relevance of examining in detail the kind of norms that are used for subjecting individuals to social control and the quality of the legal regulation. His analysis of the regulation of the residence restrictions for asylum seekers in Ceuta, Melilla and the Canary Islands reveals that these restrictions do not fulfil all the elements of legal social control. Firstly, there is no proper legal basis for the restrictions, which means that the authorities act without having been vested with the authority to prohibit asylum-seekers from moving to the mainland. A second reason is that social control is exercised solely by the executive branch, without the participation of the legislature, which has been absent since the beginning of the practice, or the courts, whose rulings have been continuously ignored by state authorities. Ruiz Ramos argues, therefore, that the practice in the enclaves could be considered a ‘pre-legal’ or a ‘diluted’ version of legal social control, which is highly problematic from the perspective of the rule of law.

Finally, and to close the circle, *Sue-Mari Viljoen's* (2025) contribution deals with a similar scenario as in Flynn's contribution: that of homeless, rough sleepers in a city, who, therefore, face the *absence* of housing. Her contribution includes a case study into the regulation of public property and rough sleeping in the city of Cape Town in South Africa, a context characterised by a very high percentage of rough sleepers that is, to some extent, normatively accepted. Viljoen analyses the (conflicting and inadequate nature of the) regulatory framework pertaining to street people. Her analysis shows how this framework is grounded in two conflicting, yet conventional approaches, that of kerbing antisocial behaviour and offering welfare-oriented types of social assistance. This is problematic as the former prevents rough sleepers from performing basic human activities, such as sleeping, washing, and urinating because they do not have a private property to perform those actions, whilst the latter fails to provide access to land or housing. She argues for an alignment between what is normatively accepted and property rules, for example, by setting aside public property for those without private property to perform private actions.
